# BCG Impact on PD-1/PD-L1 Expression in Peripheral Immunocytes of Cancer Patients—A Potential Explanation for Its Activity in Preventing Alzheimer’s Disease

**DOI:** 10.3390/cimb47080651

**Published:** 2025-08-13

**Authors:** Benjamin Y. Klein, Ofer N. Gofrit, Charles L. Greenblatt

**Affiliations:** 1 Department of Microbiology and Molecular Genetics, Hebrew University Medical School, Jerusalem 9112102, Israel; charlesg@ekmd.huji.ac.il; 2 Department of Urology, Hadassah University Medical School, Ein-Karem, Jerusalem 9112001, Israel; ogofrit@gmail.com

**Keywords:** BCG vaccine, Alzheimer’s disease, checkpoint proteins, capillary-immunoelectrophoresis, peripheral blood mononuclear cells

## Abstract

We found, retrospectively, that BCG therapy in non-muscle invasive bladder cancer (NMIBC) reduces the rate of Alzheimer’s disease. Blockade of the ligand PD-L1 or its checkpoint receptor PD-1 has been shown to improve cognitive function and reduce brain pathology features in a mouse model of Alzheimer’s disease (AD). Given that peripheral blood mononuclear cells (PBMCs) are involved in aging brain pathology and thus represent a potential AD therapeutic target, we analyzed the impact of BCG on the expression of PD-1, PD-L1, and inflammation modulators in PBMCs. Cryopreserved PBMCs pre- and post-BCG-treated six melanoma and six NMIBC patients were repurposed for immunoelectrophoretic analysis of PBMC-extracted proteins. PBMCs, post-BCG treatment in melanoma patients, were harvested only 4 months after the start of treatment (short BCG period), whereas the PBMCs of NMIBC patients were harvested 24 to 52 months after starting the BCG treatment. In melanoma PBMCs, BCG upregulated PD-L1 (*p* = 0.052) while downregulating PD-1 (insignificantly, *p* = 0.16). In contrast, in NMIBC patients, BCG downregulated PD-L1 (insignificantly, *p* = 0.67), while upregulating PD-1 (*p* = 0.0082). PD-L1 positive correlation with p-IkB (r = 0.7228) under BCG is inverted to that of PD-L1 against IkB (*p* = −0.9491). The difference between these opposite correlations is significant (*p* = 0.011), indicating that PD-L1 is upregulated early after BCG treatment, in association with p-IKB, which enables inflammation. This association subsided later, and for PD-1, did not occur at the short or long BCG periods. Experiments with a larger number of patients may substantiate the hypothesis that an increase in PD-1 by BCG relative to PD-L1 may protect against AD.

## 1. Introduction

### 1.1. Justification of Using Vaccines to Study Alzheimer’s Disease

Sporadic (non-familial late onset) Alzheimer’s disease (AD) has a heterogeneous etiology reflected by numerous genome-wide association studies (GWAS) that result in large numbers of gene products that are not mechanistically unified. GWAS of large numbers of AD patients attempting to decipher pharmacological targets may result in several different deranged gene products requiring different drugs to influence each group of deranged genes [1]. An alternative approach to identifying potential prophylactic therapies is to proceed bottom-up, retrospectively identifying an agent that prevents AD in a subset of individuals from a heterogeneous population and analyzing the response to this agent of a signaling pathway related to one of the abnormal signs of AD. We have chosen the bottom-up approach. Retrospective studies analyzing patients treated with intravesical instillation of Bacillus Calmette-Guérin (BCG) vaccine, to treat non-muscle-invasive bladder cancer (NMIBC), have shown a 4-fold decrease in the occurrence of AD compared to controls treated with other methods [2]. Subsequently, adding to the suggestion that BCG might reduce the rate of AD, it has been claimed that immunization with several other vaccines can reduce the incidence of AD compared to controls [3,4]. Such vaccination results indicate that possible protection against AD is unrelated to any specific antigen but rather to metabolic changes induced by innate [5] and adaptive immune activation [6] of immunocytes.

### 1.2. Peripheral Immunocytes Involvement in AD

The concept of activated immunocytes that protect the brain was pioneered years before our retrospective results, which revealed that peripheral immune cells interacting with the central nervous system (CNS) are therapeutic for CNS damage [7], and perhaps prophylactic for neurodegeneration [8]. Furthermore, it has been suggested that protecting against AD is not necessarily centric to the brain but rather depends on peripheral immunocytes [9]. Neurodegeneration associated with aging has been connected to brain-infiltrating immunocytes [10,11], and bone marrow-derived hematopoietic cells have been identified in brain tissue [12,13,14,15,16].

### 1.3. Repurposing of PBMCs Under BCG Vaccine Treatment for Studying Molecules Involved in AD

The retrospective finding that NMIBC patients treated with repeated sessions of intravesical BCG instillations had reduced AD has prompted experiments on the effect of BCG on peripheral blood mononuclear cells (PBMCs). The main analysis was on unfolded protein response (UPR) to endoplasmic reticulum (ER) stress signaling, in both NMIBC [17] and melanoma patients [18]. Given that blockade of the immune checkpoints in an animal model of AD has modulated the cognitive tests and brain pathology [19,20,21], we allocated PBMC protein extracts from our previously UPR-analyzed patients to evaluate the impact of BCG therapy on the expression of immune checkpoint proteins in PBMCs. We measured death protein 1 (PD-1), its ligand (PD-L1), the inhibitor of NF-kB (IkB), which is the master transcription factor of inflammation genes, and its phosphorylation-disrupted inhibitor (p-IkB). This study was designed to generate hypotheses to explain how BCG may protect against developing AD in at least part of the population destined to develop the disease. PD-1 is expressed on activated immunocytes that are capable of killing target cells via surface receptors specific for binding to surface antigens expressed by the target cells [22].

### 1.4. PD-L1 Signaling in PD-1 Target Cells

The role of PD-1 bound to its ligand, PD-L1, expressed on the target cell, was identified as a keeper of self-tolerance that inhibits autoimmunity [22]. Most research on PD-1/PD-L1 binding has been in the field of oncology, focusing on tumor-infiltrating lymphocytes (TILs), where PD-1 binds to tumor PD-L1, activating the Akt/mTORC1 signaling cascade in the PD-L1-expressing target. This has been shown to induce increased translation rate of mRNA of the glycolytic pathway enzymes, thus converting anaerobic to aerobic (increased rate of) glycolysis in tumor cells [23]. This increases glucose consumption from the extracellular fluid by the tumor, causing a relative deprivation of glucose available to TILs, and reducing TILs’ immunological efficiency due to a lack of their energy source. PD-L1 short intracellular sequence contains motifs that protect cells against interferon-induced apoptosis, provided it is in a transmembrane position [24]. PD-L1 blocking antibodies disengage the TILs’ PD-1 from the tumor PD-L1, causing its internalization, which disrupts the Akt/mTORC1 activation of high-rate glycolysis in tumor cells [25]. The blockers restore the glucose supply to starved TILs, which in turn, can kill the tumor cells without the interference of PD-L1 [23].

### 1.5. Aims of Studying PD-1/PD-L1 in PBMCS Under BCG Concerning Alzheimer’s Disease

Here, we measured the extent of positive and negative changes in the checkpoint proteins in response to BCG therapy, intending to generate a hypothesis that connects BCG with a possible mechanism that may prevent AD via PD-1/PD-L1 interaction. Part of the emphasis was on the time from therapy initiation, and it was found that the PD-1 expression increases, while PD-L1 decreases as time passes. Although these results were obtained in PBMCs, they may relate to the influence of checkpoint proteins in cognition [19] and to the presence of hematopoietic cells in the brain [12]. Therefore, these findings require further exploration.

## 2. Materials and Methods

Protein samples derived from PBMC of two groups of cancer patients (melanoma and NMIBC), treated by different protocols and exposed to two different BCG vaccine strains, each by a different route, intra-dermal and intravesical, respectively. Initially, the PBMC protein samples were analyzed for the impact of BCG on the endoplasmic reticulum (ER) stress response [17,18]. Due to the heterogeneity of humans, we used the pre-BCG PBMCs as negative controls compared with the post-BCG PBMCs for each patient.

### 2.1. Treatment Protocol

The treatment of melanoma patients (grade III-IV) described in previous studies [26,27] and reiterated for repurposing PBMCs to study their UPR signaling in response to BCG [18]. Briefly, it required the preparation of individual patients’ autologous tumor cell vaccines, which consisted of irradiated (230 Gy) metastatic cells subsequently conjugated with dinitrophenol to enhance their immunogenicity via Th1 cells. This anti-melanoma vaccine was injected intradermally (10–25 × 10^6^ cells) every 3 weeks, repeated eight times. BCG was added to the tumor cell vaccine; in distinction from the NMIBC protocol, the BCG vaccine served as an adjuvant. The BCG strain was from the Danish Statens Serum Institute and administered at a 1:50 dilution for the first three injections and 1:500 for the last five doses. Before and after this treatment course, PBMCs were isolated by Ficoll density centrifugation and cryopreserved in 1 mL growth medium, 20% fetal calf serum, and 10% dimethyl sulfoxide in liquid nitrogen until used as described below. As part of an old study, these PBMCs were kept cryopreserved for 7 to 11 years before being repurposed. Figure S1 converted proteins into virtual bands, demonstrating that time in cryopreservation is unrelated to protein quality.

Bladder cancer patients with stage Ta or T1, high-grade NMIBC, were treated by BCG therapy with six weekly intravesical instillations of OncoTICE BCG in 50 cc of normal saline. PBMC isolation was performed before and after the course of BCG therapy. Age, gender, and time of blood sampling after BCG therapy are recorded (Table 1). This study was approved by the committee on research involving human subjects of the Hebrew University Hadassah Medical School (# 0798- 21 HMO)

### 2.2. PBMC Sampling

Reagents from Sigma (Rehovot, Israel) were used to harvest, cryopreserve, and thaw PBMCs. Before and after the course of intravesical exposure to BCG, venous blood with anticoagulant (EDTA) was drawn, diluted 1:2 in Dulbecco’s modified buffered saline (#D0537), and separated on a Ficoll (Histopaque 1.077 g/mL, # 10771) density centrifugation at 1200 x g for 30 min at room temperature. PBMCs at the interphase between Ficoll interphase and the dilute plasma upper-phase were aspirated, washed in ice-cold RPMI-1640 medium (# R8758), suspended in a mixture of 10% dimethyl sulfoxide (DMSO) and 20% fetal calf serum (FCS, #F9665) in RPMI-1640 at °C degrees and gradually frozen to −170 °C, and kept in liquid nitrogen until further use.

### 2.3. Thawing of PBMC and Protein Extraction

Frozen PBMCs were thawed in a water bath at room temperature until reaching 0 °C. Cells were diluted in 20 volumes of Dulbecco phosphate-buffered saline (PBS) 2% FCS at 0 °C, spun for 10 min at 300× *g* at 4 °C, and washed twice in cold PBS to remove all cryopreservation material and FCS traces. The cell pellets were suspended in a 0.2 mL Bicine/CHAPS detergent lysis buffer kit (CBS 403) at 0 °C, containing inhibitors of proteases and phosphatases (Protein Simple, San Jose, CA), minimizing nuclear lysis. The cells were incubated on ice for 30 min and spun for 30 min at 10,000× *g* at 4 °C. Supernatants of proteins were frozen at −20 °C until further use.

### 2.4. Preparation of Immune-Electrophoresis Samples

Samples were prepared using a separation module kit designed for a molecular weight range of 12–230 MW and for a 25-capillary cartridge (SM-W004) for an Abby instrument (Protein Simple website for the Abby instrument). A master mix solution was prepared to mix the protein samples according to the producer’s instructions using a standard pack from the kit. It provides dithiothreitol, a proprietary master mix compound, and a 5x concentrated sample buffer. The samples contained 0.0006 mL ready-to-use master mix with 0.0024 mL of protein solution (total volume/sample = 0.003 mL, containing 0.0008 mg protein), boiled at 95 °C for 5 min, and loaded on a producer-provided plate in a row of 24 wells for the samples and 1 well for the producer-supplied biotinylated molecular weight ladder. The kit’s detection module (mainly DM-001 [antirabbit]) contained the secondary antibody HRP conjugate, the antibody diluent, which is also fit to serve as a blocking buffer, luminol, and hydrogen peroxide. Other parts of the separation module of the kit contained a wash buffer, plates, and capillary cartridges. We have used a total-protein detection module (DM-TP01) in a RePlex format in which, after the immune electrophoresis run, the antibodies undergo in-capillary stripping and a catalytic reagent biotinylates the total capillary proteins (in-capillary), which are detected by streptavidin. The primary antibodies are purchased from other suppliers. From loading the plate onto the Abby instrument to the end of the procedure, all the described electrophoretic and catalytic steps are automatically performed and recorded by the computer-run files, without human intervention.

### 2.5. Antibodies

Anti-PD-1 rabbit monoclonal antibodies (R-mAb) XP Cell Signaling Technologies (CST) #86163T, anti-PD-L1 R-mAb XP CST #972113684T, anti-IkBa R-mAb CST #4812, anti-phospho-IkB Ser32 R-mAb CST #2859. Antibodies were diluted 1:100 in the diluent solution provided in the detection module of the electrophoresis kit (Protein Simple).

### 2.6. Analysis of the Results

The Abby instrument presented results of protein curve peaks (detected by the primary and secondary antibodies) as protein quantities under the curves recorded in the run files. The arbitrary numbers of the antigen sizes are copied from the original run files of the instrument and presented in Supplementary Table S1. The protein peaks are expressed relative to total protein values obtained by a separate kit for in-capillary protein quantitation. The total protein in each capillary is recorded by the instrument and copied into an Excel file, by which antigen quantities from respective capillaries are normalized.

### 2.7. Presentation of the Results

The arbitrary antigen densities are presented as a fraction of the total protein density, obtained as arbitrary values of chemiluminescence, as detected by the Abby instrument. These relative antigens of signaling protein quantities are presented per patient as pre- vs. post-BCG treatment values.

#### 2.7.1. Bar Graphs of Mean Values Compared by *T*-Test

The illustration shows comparative bar graphs (comparing mean values of pre-BCG to mean values of post-BCG by Student’s *T*-test). Two conditions were compared, control vs. experimental, of the same individuals, which did not require multiple comparison corrections.

#### 2.7.2. Correlation Coefficients of Pre- and Post-BCG Values

Regression lines, which contain pre-BCG results in blue vs. post-BCG results in red. The correlation coefficients of each regression are provided by PowerPoint software, and their statistical significance is computed using SPSS (v30).

#### 2.7.3. Comparison Between Pearson Correlation Coefficients

The regression lines of the relationship between activating proteins on the *X* axis and expected responding protein substrates on the *Y* axis. For comparing regressions, correlation coefficients were converted by Fisher’s transformation into normal distributions, and the z-scores representing the coefficients were compared. Multiple comparison corrections were not required, since the same dependent parameter of the same individuals was compared between two independent parameters.

### 2.8. Presentation of the Gains and Losses of Checkpoint Proteins

The gain or loss for each checkpoint protein per patient, obtained by subtracting the post-BCG from the pre-BCG number. These differences were used for the temporal expression of PD-1 in parallel to PD-L1.

## 3. Results

**Figure 1:**
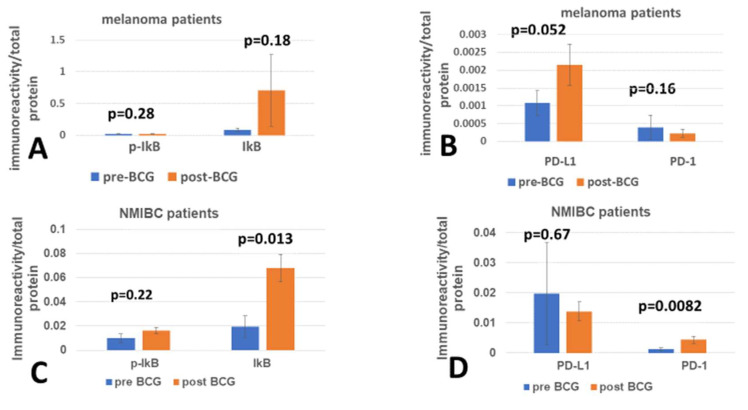
The impact of BCG on modulators of inflammation and checkpoint ligand and receptor: PBMC of two groups of six individuals each, before and after extensive exposure to the BCG vaccine, were analyzed by immunoelectrophoresis for the expression of the disrupted inflammation inhibitor (p-IkB) and inflammation inhibitor (IkB) (**A**,**C**). PD-L1 ligand and PD-1 receptor (**B**,**D**). One group of grade III melanoma patients received BCG as an intradermal adjuvant to melanoma tumor-specific antigens immunization (**A**,**B**). The second group received intravesical BCG instillations (**C**,**D**) to treat NMIBC. The interval between pre- and post-BCG treatment in (**A**,**B**) is mostly only 4 months, whereas in (**C**,**D**), it is variable, ranging from 24 to 52 months.

The checkpoint generated between the PD-1 (programmed death protein-1) expressed by immune-stimulated peripheral immunocytes and its ligand PD-L1 expressed by the tumor target cells is expected to engage the antitumor cytotoxic killer cell and its tumor target in a manner that prevents cytotoxicity [28]. In an exploratory pilot study to test the impact of the BCG vaccine on the unfolded protein response to ER stress in PBMC, the expression of the checkpoint receptor PD-1 has been tested, pertinent to the fact that these PBMCs were isolated from melanoma [18] patients and a second group of NMIBC patients [17]. Although the usual concern is that the PD-L1 ligand of the PD-1 receptor is expressed by tumor cells, it was also included in the testing of PBMCs. The modulation of inflammation by the NF-kB inhibitor (IkB) and its disruption by phosphorylation (p-IkB) in PBMCs was tested in parallel, since inflammation is potentially involved in tumor [29] and AD [30] biology. Figure 1 shows the mean expression of p-IkB, IkB, PD-L1, and PD-1 in PBMCs of pre- and post-exposure of melanoma patients (Figure 1A,B) and NMIBC patients (Figure 1C,D) to BCG treatments. The immunoreactivity of PD-L1 and PD-1 is at least one order of magnitude lower than that of IkB in melanoma patients, and so is the PD-1 level of expression in NMIBC patients, in which PD-L1 levels were much higher compared with PD-1. The pre- vs. post-BCG ratio between PD-L1 and PD-1 in NMIBC is inverted to that of the melanoma patients, which may have implications for therapy. In the melanoma group, the pre-BCG mean PD-1 level was higher than the post-BCG level, although statistically insignificant (*p* = 0.16, *n* = 6); in contrast, in the NMIBC patients, the post-BCG level was significantly higher than the pre-BCG mean level (*p* = 0.0082, *n* = 6). Interestingly, in NMIBC patients, the pattern of PD-1 change is parallel to that of IkB, meaning that with BCG treatment, although associated with inhibition of inflammation (high IkB), the PD-1 levels were upregulated.

**Figure 2:**
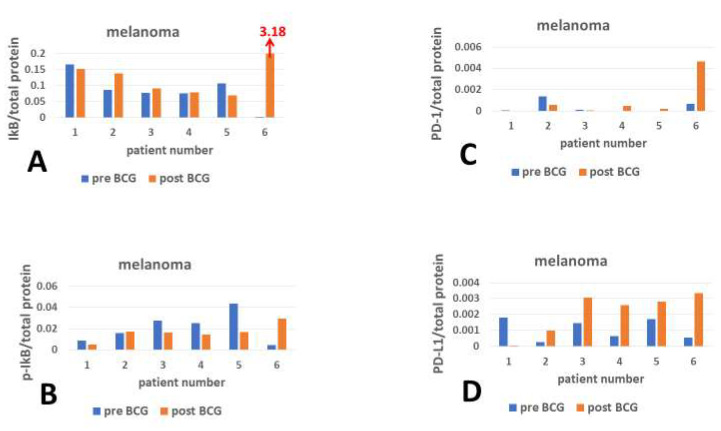

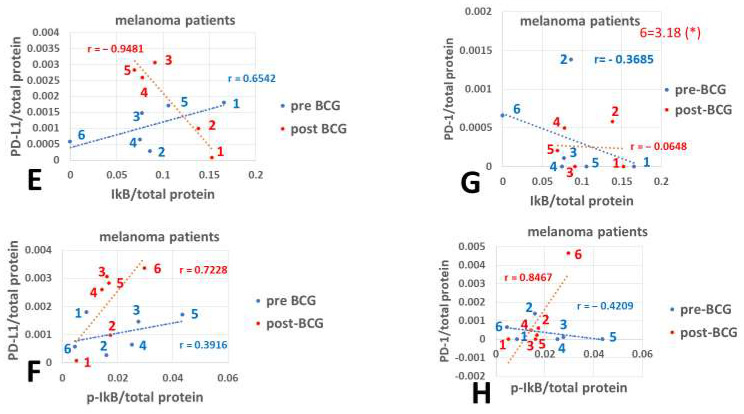
The impact of BCG-intensive vaccination on IkB inhibitor of NF-kB, its disrupted form p-IkB, in parallel to PD-1/PD-L1 checkpoint expression in melanoma patients' PBMCs: Proteins from PBMCs of six melanoma-numbered patients, before and after BCG vaccination courses, were analyzed by capillary immunoelectrophoresis, using primary antibodies against IkB, p-IkB, PD-1, and PD-L1. IkB (**A**) and p-IkB (**B**), PD-1 (**C**) and PD-L1 (**D**) are illustrated as pre-BCG (blue) side by side with the post-BCG (red) abundance per total protein. The checkpoint ligand PD-L1 expressed by PBMCs on the dependent *Y* axis is matched with IkB (**E**) and p-IkB (**F**) on the independent *X* axis. Similarly, the PBMC-expressed checkpoint receptor PD-1 is matched with IkB (**G**) and p-IkB abundance (**H**). The matching for each enumerated patient results in a dot scatter of pre-BCG (blue dots) vs. post-BCG (red dots), and regression lines are illustrated. Note that the outlier IkB post-BCG value of patient 6 (*) is excluded from the dot scatter in panels (**E**,**G**).

The high error bars in the mean levels of IkB in Figure 1A and of PD-L1 in Figure 1D necessitate presenting each individual separately. Figure 2D shows that PD-L1 is variably expressed in the PBMCs of all melanoma patients. After the intensive BCG treatment course, PD-L1 expression was upregulated in patients 2, 3, 4, 5, and 6, and downregulated in patient 1. The inhibitor (IkB) of the master transcription factor of the inflammatory gene transcription program, NF-kB, in patient 1 (Figure 2A) does not align with the substantial downregulation of PD-L1 (Figure 2D) in that same patient; however, there is a decrease in IkB disruption (p-IkB, Figure 2B, patient 1). The expression of IkB after BCG therapy relative to pre-BCG is higher only in patients 2 and 6 (Figure 2A), which aligns with the increased expression of PD-L1 in these same patients (Figure 2D). The phosphorylated (disrupted) IkB (p-IkB, Figure 2B) aligns with the increased PD-L1 expression only in patients 3, 4, and 5 (Figure 2D). Watching the bar graphs of checkpoint and IkB expressions, the relationship between inflammation markers and checkpoint protein expression is not evident, necessitating matching between these markers, patient by patient. The only clear distinction is that BCG treatment increases PD-L1 expression in patients 2, 3, 4, 5, and 6 (Figure 2D). Post-BCG, PD-1 expression is higher relative to pre-BCG only in patients 4, 5, and 6 (Figure 2C).

To measure the correlations between markers of active and disrupted inflammation inhibitors and the checkpoint protein expression, PD-L1 and PD-1 were matched with IkB and pIkB in pre- and post-BCG in PBMCs for each patient. The PD-L1 (*Y*-axis Figure 2E) matched with IkB (*X*-axis) resulted in a dot scatter in which those only under BCG treatment showed increased PD-L1 vs. pre-BCG treatment (patient 1 being the exception), and as a group, they showed a significant descending regression line (r = −0.8259, *n* = 6, *p* = 0.043), indicating that the pre-BCG dot scatter regression was insignificant. The value of IkB/total protein in patient 6 post-BCG was too distant from the IkB values of the rest of the patients, constituting an outlier (3.18 units, Figure 2A), and was excluded from the regression line, resulting in r = −0.9481 (Figure 2E). Patient 6 exhibited an excessive response to BCG regarding UPR signaling proteins [18], which may be related to an exceptional sensitivity to BCG of epigenetic remodeling enzymes. PD-L1 matched with p-IkB of the post-BCG treatment showed an ascending regression line (Figure 2F) with a correlation coefficient above the *p*-value of 0.05 (r = 0.7228, *n* = 6, *p* = 0.109). Here, 4 patients show higher PD-L1 expression than the pre-BCG values. Comparing the p-IkB (Figure 2F) with the IkB (Figure 2E) regression (red) lines against PD-L1 shows significantly different opposite trajectories (*p* = 0.011), indicating that inflammation and its inhibition (p-IkB) is associated with increased and (IkB) decreased PD-L1 expression 4 months from the start of BCG treatment, respectively (12 months of patient 5 being an exception). The expression levels of PD-1 checkpoint receptor before BCG treatment are lower than after treatment only in patients 4, 5, and 6; however, as a group, the pre-BCG PD-1/total protein expression decreases as the IkB/total protein increases (Figure 2G), contrary to the PD-L1 trajectory (Figure 2E). The inclusion of patient 6 IkB expression after BCG treatment is not shown; if it had been included, the BCG treatment would have become positive (r = 0.9902, *n* = 6, *p* = 0.00014), counteracting the inhibition of inflammation by opposing the pre-BCG direction of the regression line (blue regression line, Figure 2G). PD-1 expression vs. p-IkB (Figure 2H), which represents the proinflammatory trajectory, also shows an increase in PD-1/total protein expression after BCG treatment, in parallel with the increase in proinflammatory values of p-IkB (Figure 2H). Thus, during the early period (4 months) from the start of BCG treatment, the opposing inflammation trajectories influenced by BCG can be attributed to the opposite progression of PD-L1 growth. Contrary to PD-L1, the increase in PD-1 can be attributed to the treatment by BCG, as discerned from the BCG effect on inflammation.

**Figure 3:**
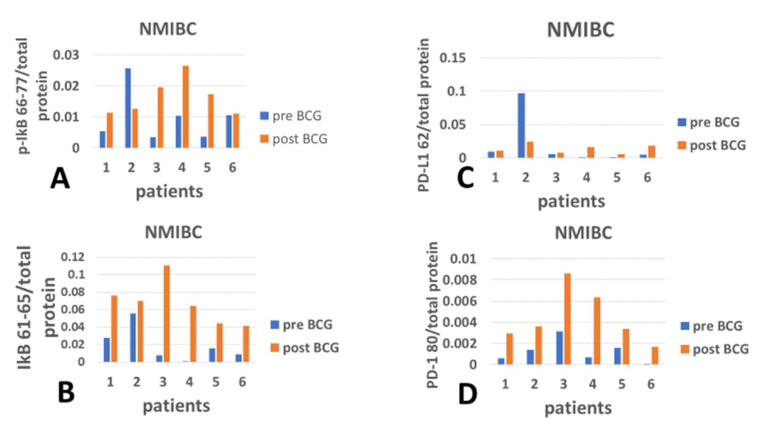

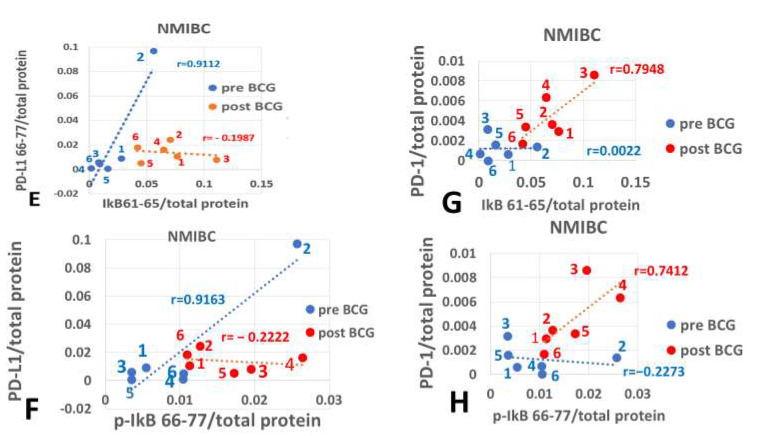
The impact of repeated bladder exposure to BCG on NF-kB active and inactivated inhibitor in parallel to PD-1/PD-L1 checkpoint expression in PBMCs of NMIBC patients: Proteins from PBMCs of six NMIBC patients, before and after BCG intravesical instillation courses, were analyzed by capillary immunoelectrophoresis, using primary antibodies against p-IkB, IkB, PD-L1, and PD-1. The peaks of p-IkB (**A**), IkB (**B**), PD-L1 (**C**), and PD-1 (**D**) are illustrated pre-BCG (blue) side by side with the post-BCG (red) abundance per total protein. PD-L1 matched with IkB (**E**) and pIkB (**F**) to produce a dot scatter of pre-BCG (blue) and post-BCG (red). The correlation coefficients of pre-BCG (**E**) are r = 0.9112, and the post-BCG, r = −0.1987; the difference between their opposite direction is significant (*p* = 0.033, *n* = 6). PD-L1 matched with pIkB (**F**) shows the pre-BCG correlation of r = 0.9163 and post-BCG, −0.2222; the difference between their opposite directions is significant (*p* = 0.028, *n* = 6). PD-1 is presented as dot scatters against IkB (**G**) and pIkB (**H**), and their post-BCG regression lines are similar, r = 0.7948 and r = 0.7412, respectively, and thus, similarly correlate with anti-proinflammation.

Figure 3 displays the checkpoint and inflammation modulation response of PBMCs to intravesical exposure to BCG of NMIBC patients. Here, there is an exceptional response by patient 2 regarding PD-L1 vs. p-IkB, but it is similar to that of patient 1 in the melanoma group vs. IkB. In contrast to the melanoma group, PD-L1 expression increased congruently with the increase in p-IkB in patients 1, 3, 4, 5, and 6 (Figure 3A vs. Figure 3C). Here, BCG induced an increased expression of p-IkB, unlike the lack of response in the melanoma group (Figure 2B). The expression of the cognate receptor of PD-L1, PD-1, responded to the BCG therapy in all 6 patients (Figure 3D). Both p-IkB (Figure 3A) and IkB (Figure 3B) show increased expression in response to BCG therapy, which conflicts with the opposite function of these isoforms. The correlation of IkB and p-IkB with PD-L1 under BCG is disrupted (becomes almost a flat line), *p* = 0.1987 (Figure 3E) for IkB and 0.2222 (Figure 3F) for p-IkB, respectively. Also, when IkB and p-IkB are matched with PD-1, both show an increase (see *X*-axis, Figure 3G,H) in PD-1 on the *Y*-axis. The increased abundance of both PD-L1 and PD-1 under BCG therapy, with opposing inflammation markers, indicates that the effect of BCG treatment on these checkpoint proteins is independent of inflammation.

**Figure 4:**
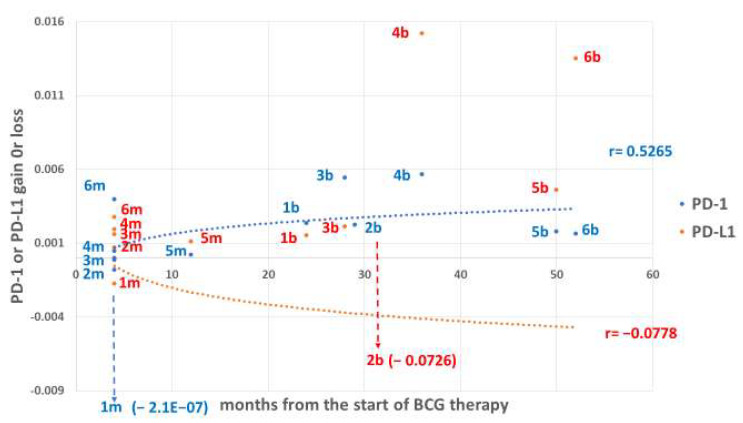
The gain or loss of PD-L1 and PD-1 expression in PBMCs in response to BCG as a function of time: Dot illustration of the PD-1 death protein (blue) and its ligand PD-L1 (red) positioned on the *X*-axis indicating time in months from the start of exposure to BCG, plotted against the gain or loss of expression in PBMCs on the *Y*-axis. The checkpoints (receptor/ligand) pairs are indicated by patient number, and the melanoma (m) and NMIBC (b) therapy groups are displayed, respectively, in months post-BCG. The *Y*-axis covers the post-BCG therapy gain, whether gain or loss of checkpoint protein expression. Note the tendency of PD-1 expression after treatment, in the melanoma (short periods), to increase above PD-L1 only in patient 6. In the longer periods of NMIBC, in three out of six patients, the expression of PD-1 is inverted to that of PD-L1. The log trajectories of gain vs. loss of expression show a positive (gain under BCG, r = 0.5265, *n* = 12) for PD-1, and a negative expression (loss) for PD-L1 (r = −0.0787, *n* = 12). Although both regression lines are positioned across the 0-line indicated by the *Y*-axis, the difference between them is insignificant (*p* = 0.17).

Figure 4 illustrates the impact of BCG on the position of the checkpoint receptor PD-1 vs. the ligand PD-L1 in each patient, for both treatment groups. The resulting expression gain (positive) or loss (negative) shows the post- vs. pre-BCG difference in each protein in PBMCs, expressed as the expression level (per total protein) of the post-BCG value. If peripheral immunocytes (PBMCs) can access the brain and interact protectively with the glia, presumably one of the preferred interactions will occur between immunocytes expressing the receptor PD-1 and a glial cell expressing the ligand PD-L1. Immunocytes within the PBMCs expressing PD-L1 ligands and competing for PD-1 moieties expressed by PBMCs may neutralize PD-1-expressing immunocytes before they access the brain. The distance between the abundant PD-L1 and the scarce PD-1 expression reflects the inhibitory potential of PD-1 cells by PD-L1 cells within the PBMCs, peripherally, before reaching the glial cell targets. The distance hierarchy of PD-L1 gain over PD-1 is: patient 4 > 3 > 2 > 5 > 1 for the melanoma treatment group, where patient 6 is the exception in which PD-1 ended with a higher expression gain than PD-L1. The time interval from the start of therapy to the analysis is only 4 months (12 months for the melanoma patient 5). The tumor cells are not part of this study, yet it may be estimated that if immunocytes expressing PD-L1 are in surplus over PD-1-expressing immunocytes, the latter will be neutralized or impeded by the former cells. This way, immunocytes that lack PD-1 (or have it blocked) will be set free to lyse tumor cells even if those express the PD-L1 ligand by avoiding the checkpoint inhibitory effect. (This assumption is valid only if the antitumor immunocytes are not TILs energetically competed out by the glucose-consuming tumor). According to these thoughts, advancing brain wellness is opposed to cytolyzing the melanoma tumor cells. However, the time interval provided for the BCG therapy of the melanoma patients in Figure 4 is too short, considering the time-dependent changes observed for BCG therapy in the NMIBC patients (“b” patients in Figure 4). The gain or loss of expression shows the extent to which BCG advanced the abundance of PD-1 vs. the lower abundance of PD-L1. According to the gain levels, PD-L1 of the melanoma patients 1, 2, 3, 4, and 5 (but not patient 6) advances faster than PD-1 growth at the 4-month time point. After longer time intervals, the rate of PD-1 abundance in PBMCs of NMIBC patients 1, 2, and 3 is inverted compared with melanoma patients 1, 2, 3, 4, and 5 (Figure 4, “b” patients). The time effect is consistent with the time required for BCG to show its impact on several functions, such as anaerobic conversion to aerobic glycolysis [31], and the progression in T cell surface antigen expression [32], both shown to be time-dependent post-BCG treatment.

## 4. Discussion

### 4.1. Analysis of PBMCs Justified Based on Previous Studies

The reduction in AD incidence among bladder cancer patients treated with BCG [2,33] is the rationale for analyzing the impact of BCG on the PBMC functions underlying the heterogeneous AD etiology. Previously, we have shown the influence of BCG on the UPR, which processes protein aggregates in PBMCs and is expected to protect brain tissue whose survival is endangered by aggregates. The UPR was enhanced by intensive BCG treatment of NIMBC patients by intravesical BCG instillations [17] and in melanoma patients by intradermal BCG vaccination [18]. We have also demonstrated the BCG impact on the GAPDH biomarker of glycolysis. Although the UPR and GAPDH responses were less prominent in the melanoma patients, these functions in PBMCs were demonstrated to react to BCG despite the highly different variables represented by the melanoma vs. the NMIBC therapy groups. In both study groups, BCG enhanced the UPR, raising the question of whether UPR is part of innate immune training. The validity of analyzing PBMCs derives from the assumption that natural protection against AD is not necessarily centric to the brain tissue [9,34], which legitimizes analyzing peripheral immunocytes [8,35,36]. According to animal [12] and human [13,14,15,16] studies, it is likely that peripheral immunocytes originating in the bone marrow infiltrate the brain. In the present study, we examined an additional function proposed as underlying AD prevention, the increase in molecular factors responding to BCG treatment, which may also be part of the immunological activation.

### 4.2. Checkpoint Proteins’ Relation to Inflammation Inhibition

#### 4.2.1. PD-L1 Versus Inflammation Inhibitor

Recent studies have suggested that the blockade of the immune checkpoint PD-1/PD-L1 is worth targeting to protect the brain against tissue damage characteristic of AD [20,21,37,38]. The results of pre- vs. post-BCG therapy of the PD-1 and PD-L1 per total protein are compared in both groups of patients (melanoma and NMIBC). The mean PD-1 level shows a decrease under BCG therapy in the melanoma group, opposite to the pattern of the mean increase in the inflammation inhibitor IkB, although the changes in both IkB and PD-1 are not statistically significant. These demonstrate the limitation of the short time interval between pre- and post-BCG courses (only 4-month intervals in the melanoma group). Yet, in both groups, the BCG treatment can be credited for influencing PD-1 expression more than the BCG modulation of the inflammation markers (Figure 2G,H and Figure 3G,H). Note that the post-BCG regression would have been strongly opposite to the pre-BCG had the value of melanoma patient 6 been included. In contrast to PD-1, PD-L1 decreases in association with the increase in inflammation inhibitor (IkB) while increasing with the disruption of the inhibitor (p-IkB) under BCG (Figure 2E vs. Figure 2F, respectively) in the melanoma group. Thus, for the melanoma group, the impact of BCG on PD-L1-expressing PBMC can be concluded as being affected (increased) in association with failure to inhibit inflammation.

#### 4.2.2. PD-1 Versus Inflammation Inhibitor

This effect of inflammation is not shown by the PD-1 analysis (Figure 2H vs. Figure 2G). Unlike the melanoma group, the bladder cancer (NMIBC) group was treated by intravesical instillation and analyzed after longer time intervals from the start of therapy. As seen in Figure 3E vs. Figure 3F for the PD-L1 expression, and Figure 3G vs. Figure 3H for the PD-1 expression post-BCG, there are no opposing differences between IkB inflammation inhibitor and its inhibition-disrupted form, p-IkB. Thus, the increased PD-1 and PD-L1 expressions are unlikely to be credited to the inflammatory effects of BCG.

### 4.3. Temporal Requirement for BCG to Change PD-1/PD-L1 Expression Levels

Perhaps this difference between the two patient groups may be ascribed to the long interval from the start of the BCG course to the post-BCG sampling, during which the PBMC samples of the NMIBC patients were analyzed. Interestingly, the PBMCs of melanoma patient 5 were sampled 12 months after BCG therapy, unlike the 4-month interval of the rest of the group, and have already shown a reversal, increasing the rate of PD-1 expression vs. PD-L1. This difference in patient 5 is seen in Figure 2C, where PD-I increases from practically zero expression level to a detectable level that can be considered a high level of growth. In contrast, PD-L1, which showed a substantially detectable pre-BCG level, had less than 100% growth in Figure 2D. PD-L1 expressing PBMCs can be attributed to interferon gamma secreted by PD-1 expressing cells, some of which may be Th1-cells [39] designed to kill PD-L1 expressing PBMCs.

### 4.4. Hypothetical Mechanism by Which PD-1/PD-L1 Protects Against AD

PD-1-expressing PBMCs from patients, in which the anti-tumor therapy is designed to harness PBMCs to kill tumor cells, which inhibit their killing if they express PD-L1. Overcoming this inhibition of tumor killing requires blocking PD-1/PD-L1 binding. Intuitively, if our hypothesis proves valid, the neurologist’s contrasting aim is to protect brain cells hypothetically by binding PD-1-expressing PBMCs to PD-L1-expressing brain cells, thus protecting them. Yet, when a mouse AD model was treated by PD-L1 blockade, it improved cognitive function [20,21], which at first glance seems unexplainable. However, the blockers in these mice were injected intraperitoneally, which may explain their positive effect on cognition by preventing the PD-L1-expressing peripheral immunocytes from inhibiting the peripheral PD-1 cells from reaching the brain cells. It is necessary to exclude the possibility that maximal PD-1-unblocked PBMCs engage with PD-L1-expressing brain cells to protect them, by the same token that PD-1 killers end up protecting instead of killing the PD-L1-expressing tumor cell target. When PD-1/PD-L1 blockers are used for anti-tumor therapy, neuropathic signs may appear [40]. This could be explained by PD-L1 blockers reaching neurons and brain cells as a spillover from their intended PBMC target. The PD-L1 expression on PBMCs is known to respond to BCG via IFNγ [41], which may raise worries about the safety of BCG alone without a PD-L1 blocker. This may be the case if the follow-up of PBMC is performed too early after the initial exposure of patients to BCG, as in the melanoma group described in Figure 4, where almost all patients (five out of six) showed a higher growth rate of PD-L1 than PD-1 PBMCs. Contrarily, the NMIBC group showed the opposite: in three out of six patients, the PD-1 percent growth was higher than that of PD-L1. The dependence of the beneficial BCG effect on a prolonged time interval of up to 3 years has been shown for the epigenetic demethylation remodeling in T-reg cells. Demethylation progressed gradually, year by year, during 3 years of 11 Foxp3-positive T cell genes, presumably gaining suppressive capabilities against diabetogenic T cells in insulin-dependent diabetes patients [42]. Beyond the biology of PBMCs under BCG, in the NMIBC patients, the bladder tumor expresses PD-L1 under BCG therapy [43]; therefore, the urologist’s advice is to add a PD-L1 blocker to the BCG treatment [44]. The studies that showed a reduction in AD incidence in NMIBC patients [2] preceded the realization that PD-L1 blockade may be required to support successful BCG treatment against bladder cancer. How, despite BCG activation of PD-L1 in PBMCs, did the BCG vaccine alone succeed in protecting against AD? Part of the answer may be explained by the finding that melanoma patients treated with an autologous tumor vaccine combined with BCG develop T cell activities against autologous tumor cells and, surprisingly, against their autologous PBMCs [45], especially after long follow-up. Long ago, we observed that the response to lymphoma antigen immunization with isolated lymphoma antigens may cross-react with normal lymphocytes [46,47]. This raises the question of whether tumor-stimulated killer cells preferentially neutralize PD-L1-expressing PBMCs, thereby freeing PD-1-expressing PBMCs to engage in brain cell protection and inhibit AD development. Such a phenomenon has been shown to occur against PD-L1-expressing tumor cells that present PD-L1 epitopes by surface HLA molecules [48,49,50]. The BCG-treated NMIBC PBMCs in Figure 4 may support the idea that, upon prolonged follow-up, there is indeed a preferential gain in PD-1-expressing PBMCs in three out of six patients over that of PD-L1-expressing PBMCs. There is one interesting result that was not followed biochemically in our NMIBC patients, other than those tested in the present study, which suggests that BCG therapy improved cognition test results [51]. The linkage between improved cognition and the involvement of PD-1 remains hypothetical until proven or disproven. Some are performed within a period similar to that of the present study. It may be concluded that BCG, as a single agent, activates a range of gene products that cover sufficient biochemical and immunological functions in protection against AD.

### 4.5. Limitations of This Study

The performance of the present research was conducted under several limitations. Firstly, in the case of melanoma, it was unlikely to convince normal controls to undergo the harsh treatment of vaccination with melanoma-tumor-specific antigens, let alone with the addition of multiple BCG vaccinations used as adjuvants. This constraint left us with the one-time possibility to repurpose cryopreserved pre- and post-BCG treatment PBMCs, and limited us to six patients and a minimal amount of clinical information. Secondly, still in the case of melanoma, the four-month short interval from the start of BCG treatment was a fixed and given time interval. The nature of BCG’s cumulative functional expression is a limitation per se. In the case of NMIBC, it was even less likely to convince normal controls to undergo intravesical BCG instillations. Another limitation is the lack of normal (non-patients) untreated with BCG, whose inclusion would have required many dozens of individuals due to the enormous heterogeneity of the human immunogenetic gestalt. This lack of normal controls was solved by pre-BCG results paired with untreated cancer patient controls for each patient. The lack of cancer patients untreated with BCG would not fulfill as a control cancer group that has not been treated with BCG because this kind of control will not compare with BCG-treated cancer patients better than the pre-BCG vs. post-BCG within the same patients. The fact that protection against AD is studied in cancer patients may distinguish them from non-cancer patients regarding developing AD under the tumor, which challenges the PD-1 expressing PBMCs. BCG conversion of PD-1-expressing immunocytes from anaerobic to aerobic glycolysis may improve their competitiveness with PD-L1-expressing tumors for extracellular glucose. Perhaps the ethical constraint on studying BCG effects in AD only in cancer patients has an advantage and constitutes a counter-limitation vs. the above considerations. One may claim that vaccination with melanoma antigens and the resulting interaction of PD-1-expressing immunocytes among the responding cells constitute a confounding factor that would not exist had experiments been conducted in non-cancerous patients. The low level of PD-1-expressing PBMCs at the early period of anti-melanoma treatment could be explained by engagement of most PD-1-expressing PBMCs by the intratumor environment due to binding to tumor PD-L1, which would have been freed had we obtained late-stage PBMCs. However, we are unlikely to obtain late-stage PBMCs from such protocols.

### 4.6. Conclusions and Perspectives

The modulation of cognition by PD-I/PD-L1 blockers administered peripherally [19,20] prompted us to test the expression of PD-1/PD-L1 in peripheral immunocytes (PBMCs) under BCG treatment, which enhanced cognition [2,51]. Early effects of BCG increased PD-L1, opposite to the decrease in PD-1, whereas late BCG effects decreased mean PD-L1, opposite to the increase in mean PD-1. Only the (early) increase in PD-L1 and the late increase in PD-1 were significant, yet the inverted patterns are compelling. We hypothesize that the inverse temporal changes in PD-1 vs. PD-L1 under BCG may be responsible for the observed improved cognition. The outlook for the future is to increase the number of studied NMIBC patients because it is worthwhile to repurpose their immunocytes that are exposed to intense BCG therapy. It will be of interest to determine if there are interactions between peripheral immunocytes via PD-1/PD-L1 and the quantitative dynamics at early vs. late post-BCG periods. New methods are available to demonstrate surface molecules responsible for peripheral immunocyte binding and a complete landscape of epigenetic changes under intense BCG therapy beyond already known immunologically functional genes. Moreover, cooperation with groups that study brain tissue is necessary for repurposing histological sections for the identification of peripheral immunocytes physically interacting with brain cells, and to identify surface molecules responsible for such interactions.

## Figures and Tables

**Table 1:** Donors of PBMCs before and after BCG therapy.

		Patient Number					
		1	2	3	4	5	6
Melanoma	Gender	Male	Male	Male	Male	Female	Male
	Age	72	76	63	44	18	70
	Months from the start of BCG	4	4	4	4	12	4
Non-muscle invasive bladder cancer (NMIBC)	Gender	Male	Male	Male	Male	Male	Female
	Age	69	60	66	50	65	86
	Months from the start of BCG	24	29	28	36	50	52

## Data Availability

The data used in this study are available in the Supplementary Section.

## Supplementary Materials

The following supporting information can be downloaded at: https://www.mdpi.com/article/10.3390/cimb47080651/s1.
